# Rotavirus NSP1 Inhibits NFκB Activation by Inducing Proteasome-Dependent Degradation of β-TrCP: A Novel Mechanism of IFN Antagonism

**DOI:** 10.1371/journal.ppat.1000280

**Published:** 2009-01-30

**Authors:** Joel W. Graff, Khalil Ettayebi, Michele E. Hardy

**Affiliations:** Veterinary Molecular Biology, Montana State University, Bozeman, Montana, United States of America; North Carolina State University, United States of America

## Abstract

Mechanisms by which viruses counter innate host defense responses generally involve inhibition of one or more components of the interferon (IFN) system. Multiple steps in the induction and amplification of IFN signaling are targeted for inhibition by viral proteins, and many of the IFN antagonists have direct or indirect effects on activation of latent cytoplasmic transcription factors. Rotavirus nonstructural protein NSP1 blocks transcription of type I IFNα/β by inducing proteasome-dependent degradation of IFN-regulatory factors 3 (IRF3), IRF5, and IRF7. In this study, we show that rotavirus NSP1 also inhibits activation of NFκB and does so by a novel mechanism. Proteasome-mediated degradation of inhibitor of κB (IκBα) is required for NFκB activation. Phosphorylated IκBα is a substrate for polyubiquitination by a multisubunit E3 ubiquitin ligase complex, Skp1/Cul1/F-box, in which the F-box substrate recognition protein is β-transducin repeat containing protein (β-TrCP). The data presented show that phosphorylated IκBα is stable in rotavirus-infected cells because infection induces proteasome-dependent degradation of β-TrCP. NSP1 expressed in isolation in transiently transfected cells is sufficient to induce this effect. Targeted degradation of an F-box protein of an E3 ligase complex with a prominent role in modulation of innate immune signaling and cell proliferation pathways is a unique mechanism of IFN antagonism and defines a second strategy of immune evasion used by rotaviruses.

## Author Summary

Cells respond to virus infection by inducing a pattern of gene expression regulated by interferon (IFN) that modulates the host immune response. In order to successfully replicate, viruses have evolved mechanisms to block the induction or function of IFN and IFN-regulated genes. Multiple proteins are activated in the cell when virus infection is detected, and it is these steps upstream of IFN synthesis that are commonly inhibited by virally encoded proteins. In this study, we investigated the function of a rotavirus protein known to block IFN responses. Rotaviruses cause life-threatening gastroenteritis in infants and young children and are responsible for substantial morbidity and mortality worldwide. Here we show that the rotavirus protein NSP1 blocks the function of cellular protein β-TrCP by inducing its degradation. β-TrCP is a component of a multisubunit complex important in the proteasome degradation pathway and is required for activation of transcription factor NFκB, which is necessary for expression of IFN and IFN-regulated genes. This is a unique strategy for viral evasion of host immune responses. Identification of viral IFN antagonists is important to understanding how best to induce cellular responses that can override viral evasion strategies and help reduce spread of infection in the host.

## Introduction

Research into mechanisms by which viruses evade host defense has received increased attention in the past several years because of the potential to develop attenuated vaccines based on viruses with weakened evasion strategies, or antiviral therapies that target specific immune system antagonists. Most, if not all, viruses encode proteins that interfere with signal transduction pathways involved in induction or amplification of the immune response, particularly the innate response driven by type I interferon (IFNα/β) [1]. IFNα/β are cytokines that stimulate expression of genes that interfere directly with steps in the virus replication cycle, and genes whose protein products modulate and recruit the adaptive immune response. The antiviral state that is established by the IFN system serves to restrict virus replication and spread while effectors of the slower adaptive immune response are recruited to the site of infection.

The mechanisms of IFNα/β induction are relatively well understood [2] although proteins involved in this pathway continue to be identified [3],[4],[5]. Induction of IFNβ transcription occurs through assembly of transcription factors interferon regulatory factor 3 (IRF3), NFκB, and ATF2/c-Jun on the positive regulatory domain enhancer element of the IFNβ promoter, and the interferon stimulated response element (ISRE) in promoters of a subset IFN-stimulated genes (ISG) [6],[7],[8]. Secreted IFNβ binds to cell surface type I IFN receptors and activates the JAK/STAT pathway, resulting in formation of IFN-stimulated gene factor-3 (ISGF3). ISGF3 is a heterotrimeric complex consisting of STAT1, STAT2, and IRF9. ISGF3 translocates to the nucleus and induces transcription of IFNα and numerous ISGs, thus amplifying the response through a positive feedback mechanism. Each of the steps in the IFN signaling pathway has been reported as targets for viral IFN antagonists. A recent summary of mechanisms viruses use to block the IFN response clearly illustrates that some viral proteins target more than one step in the pathway (e.g. influenza virus NS1) and some viruses encode more than one IFN antagonist (e.g. paramyxovirus V, C, and N proteins), exemplifying the importance of IFN signaling to host defense [1],[9].

Signal transduction pathways that activate IRF3 and NFκB have been well studied in the context of the virus-induced IFN response. Upon virus detection, IRF3 is phosphorylated at its C terminus by kinases TBK1 or IKKε [10], dimerizes and then translocates to the nucleus to assemble at the IFNβ promoter and select ISRE-containing promoters in cooperation with transcription co-activators [8]. IRF3 activation is required for IFNβ transcription, and the number of reports of viruses that interfere directly with the function of IRF3, or the steps preceding its activation has grown significantly in recent years [1]. For example, the hepatitis C virus NS3-4A protease complex cleaves TLR3 adaptor protein TRIF, blocks RIG-I signaling and cleaves adaptor protein IPS (also known as MAVS/Cardif/VISA) to release it from the mitochondrial membrane, consequently blocking IRF3 activation [4],[11]. One of the more recently defined viral IFN antagonists is rotavirus nonstructural protein NSP1. NSP1 targets IRF3, IRF5, and IRF7 for proteasome-dependent degradation early post-infection [12],[13],[14]. By targeting IRFs for degradation, NSP1 enhances rotavirus cell-to-cell spread *in vitro* [12].

NFκB is required for induction of IFNβ transcription, although exceptions to the requirement for NFκB under select experimental conditions have been reported [15],[16]. NFκB is activated by numerous stimuli, and in addition to regulating gene expression associated with innate and adaptive immune responses, functions in signaling pathways that control cell division and apoptosis [17]. NFκB subunits are held inactive in the cytoplasm by association with inhibitors of κB (IκB). Phosphorylation of IκB by IκB kinases (IKKα/β) results in its rapid ubiquitination by the E3 ligase Skp1/Cul1/F-box complex, SCF^β-TrcP^, and subsequent degradation by the 26S proteasome [18]. IκB degradation releases the p50/relA (p65) heterodimer, which translocates to the nucleus for promoter binding and transcription of NFκB target genes. Multiple steps in the NFκB pathway are targets for viral IFN antagonists, some of which are common to the IRF3 activation pathway [19].

Rotaviruses cause severe gastroenteritis in infants and young children, and in newborns of most mammalian species [20]. The rotavirus genome consists of 11 segments of dsRNA encoding six structural proteins and six nonstructural proteins. Nonstructural protein NSP1 is the least conserved protein among different strains; however, a zinc-binding domain comprised of a specific pattern of cysteine and histidine residues near the amino terminus is invariant [21]. Results from our laboratory and others have shown that NSP1 from several different rotavirus strains targets IRF3 for proteasome degradation early post-infection. Expression of NSP1 in the absence of infection directs IRF3 degradation, indicating it is the sole viral protein that mediates this effect. NSP1 of simian rotavirus also directs proteasome-mediated degradation of IRF5 and IRF7 [13]. Investigation into the properties of NSP1 responsible for IRF3 degradation showed the conserved zinc finger domain is required for this activity [14].

Modification of eukaryotic proteins with ubiquitin (Ub) prior to proteasome degradation requires an E1 activating enzyme, E2 conjugating enzyme, and an E3 ligase that transfers Ub from E2 to the target substrate [22]. E3 ligases fall into two major classes of proteins that contain either a catalytic HECT domain or a RING domain. Typical RING domains consist of cysteine and histidine residues spaced in a C3HC4 pattern that coordinately bind two zinc ions [23], yet evidence is accumulating that variations of the C3HC4 pattern exist in the RING superfamily [24]. Viral proteins with cysteine-histidine rich zinc binding domains have demonstrated E3 ligase activity, and many of the cellular targets of viral E3s are associated with regulation of immune responses to infection [25]. The pattern of cysteine and histidine residues in the zinc finger motif of NSP1 is similar, but not identical, to RING finger domains in cellular E3 ubiquitin ligases. Thus we proposed that NSP1 contained a variant RING domain signature and functioned as a viral E3 ubiquitin ligase that targets proteins with roles in the IFN signaling pathway as substrates for proteasome degradation [14].

We recently reported that IRF3 was activated and stable in cells infected with porcine rotavirus strain OSU [14]. Therefore, we sought to determine if an alternative mechanism to block induction of IFNβ was encoded in the rotavirus genome. The studies reported herein revealed that NFκB activation was blocked in OSU infected cells due in part to stabilization of phosphorylated IκBα. Dissection of the steps involved in IκBα degradation revealed that the substrate recognition protein, β-transducin repeat containing protein (β-TrCP), of the cellular E3 ubiquitin ligase complex SCF^β-TrcP^ is targeted for proteasome degradation by NSP1. The SCF^β-TrCP^ E3 ligase is responsible for ubiquitination of IκBα [26],[27], thus providing an explanation for IκBα stabilization and the lack of NFκB activation in infected cells. Targeted degradation of a cellular F-box protein required for E3 ligase substrate recognition is a novel IFN evasion strategy, and defines a second mechanism of interference with the innate immune response encoded in the rotavirus genome.

## Results

### IRF3 accumulates in the nucleus of OSU infected cells

**Figure 1:**
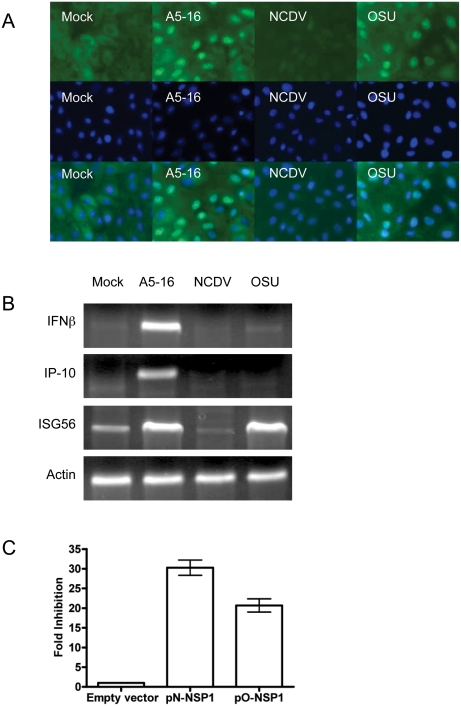
Induction of IFNβ is blocked during OSU infection, and the block is mediated by NSP1. (A) Subcellular localization of IRF3 in MA104 cells infected with three pfu/cell of the indicated virus strain was determined six hours post-infection (hpi) by IF microscopy (40×, NA 0.75). Fixed cells were stained with anti-IRF3 monoclonal antibody followed by Alexa Fluor 488-conjugated goat anti-mouse IgG. Nuclei were visualized by DAPI staining. (B) Induction of IRF3-regulated genes was analyzed by RT-PCR using RNA collected eight hpi from MA104 cells infected with three pfu/cell of the indicated virus strain. Actin mRNA was used as a template loading control. (C) 293-TLR3 cells were co-transfected with pN-NSP1 (NCDV) or pO-NSP1 (OSU), Renilla luciferase reporter plasmid phRL-CMV (Promega) and IFNβ-Luc. Forty hours post-transfection, the cells were treated with 100 µg/mL polyI:C , and lysates were prepared eight hours post-treatment. Reporter activity was measured with the Dual Luciferase Assay System (Promega). Data are represented as fold-inhibition of IFNβ reporter induction normalized to cells transfected with empty expression vector. Error bars indicate the SEM.

IRF3 is phosphorylated in OSU infected MA104 cells, indicating that the virus is detected and signaling pathways leading to IRF3 phosphorylation are intact. Once phosphorylated, IRF3 dimerizes and translocates to the nucleus to assemble with transcription co-activators on the IFNB promoter as well as ISRE-containing promoters. To test whether these latter steps in the activation pathway were affected by OSU infection, the cellular distribution of IRF3 was analyzed by IF microscopy (Figure 1A). Bovine rotavirus strains A5-16 and NCDV also were analyzed in these experiments for comparison with OSU because the behavior of these strains with respect to IRF3 activation has been described. A5-16 encodes a truncated NSP1 [28], and IRF3 is phosphorylated and stable in A5-16 infected cells [14]. NCDV encodes an NSP1 that binds to and targets IRF3 for proteasome degradation [14],[29]. The microscopy revealed that IRF3 was diffusely distributed in the cytoplasm in mock infected cells. In A5-16 infected cells, IRF3 accumulated in the nucleus as expected. IRF3 also accumulated in the nucleus of OSU infected cells, indicating nuclear translocation is not inhibited in MA104 cells infected with this rotavirus strain. IRF3 was not detected at all in NCDV infected cells, consistent with its degradation early post-infection.

### IFNβ transcription is inhibited in OSU infected cells, and the block is mediated by NSP1

The presence of nuclear IRF3 led us to test whether IFNβ mRNA was synthesized in cells infected with each of the three strains. IFNβ was strongly induced in A5-16 infected cells as expected, but not in NCDV or OSU infected cells (Figure 1B). The lack of IFNβ mRNA in NCDV infected cells can be explained by degradation of IRF3, but nuclear IRF3 in OSU infected cells suggested an alternative mechanism was used by this virus to block IFNβ transcription.

NSP1 encoded by rotavirus strains analyzed thus far directs proteasome-mediated degradation of IRF3 when expressed in the absence of infection. To determine whether NSP1 encoded by OSU played a role in inhibiting IFNβ transcription, 293-TLR3 cells [39] were transfected with a plasmid encoding OSU NSP1 (pO-NSP1) or NCDV NSP1 (pN-NSP1), and a reporter plasmid encoding firefly luciferase under control of the IFNβ promoter. Cells then were treated with polyI:C to induce reporter gene expression. Expression of OSU NSP1 inhibited polyI:C-induced luciferase expression by ∼20-fold compared to cells transfected with empty vector (Figure 1C). Reporter expression in cells expressing NCDV NSP1 was inhibited ∼30-fold; a substantial portion of this reduction can be attributed to the lack of IRF3. We note that the basal levels of luciferase expression induced by each plasmid in the absence of polyI:C treatment showed a similar pattern; that is, luciferase expression was inhibited when cells expressed NSP1, and the magnitude of the reduction was most prominent for OSU NSP1. These results demonstrate that OSU NSP1 inhibits IFNβ promoter-driven gene expression in the absence of infection, and without an obvious mechanism to interfere with the function of IRF3.

Recent studies defined a hyperphosphorylated form of IRF3 that translocates to the nucleus but is degraded, and therefore is transcriptionally inactive [30]. The transcriptional activity of nuclear IRF3 was examined by RT-PCR of IP-10, ISG56, and IFNβ mRNA. IRF3-regulated genes IP-10 and ISG56 have different transcription factor requirements for expression. While IFNβ and IP-10 generally require both IRF3 and NFκB with noted exceptions [15],[16], IRF3 is sufficient to induce ISG56 [31]. IP-10 and ISG56 mRNA were analyzed by RT-PCR to further dissect the activity of transcription factors required for expression of these genes in infected cells. A5-16 induced transcription of both IP-10 and ISG56, but neither were induced in NCDV infected cells (Figure 1B). In contrast, OSU infection induced ISG56, but not IP-10. The fact that ISG56 was induced in OSU infected cells confirmed that nuclear IRF3 is transcriptionally active. Likewise, the lack of IP-10 induction suggested OSU may encode a mechanism to block activation of NFκB.

### NFκB activation is inhibited in OSU infected cells, and the block is mediated by NSP1

**Figure 2:**
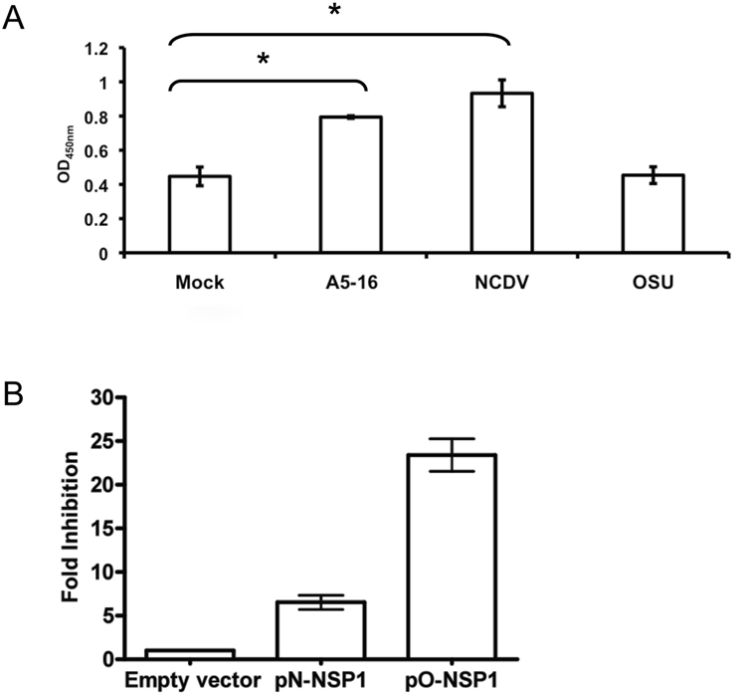
Inhibition of NFκB activation in OSU infected cells is mediated by NSP1. (A) MA104 cells were infected with 10 pfu/cell of the indicated virus strain, and cell lysates were prepared six hpi. NFκB subunit p50 binding to κB sites was determined by the TransAM p50 ELISA. Data are represented as the mean OD_450 nm_ value from three independent experiments. Error bars indicated the SEM. *p*-values were determined by unpaired Student's *t*-test (asterisk = p<0.01). (B) 293-TLR3 cells were transfected with pN-NSP1 (NCDV) or pO-NSP1 (OSU), Renilla luciferase reporter plasmid phRL-CMV, and pNFκB-Luc *Cis*. Forty hours post-transfection, cells were treated with 100 µg/mL polyI:C for eight hours, and then reporter activity in the lysates was measured with the Dual Luciferase Assay System. Data are represented as fold inhibition of NFκB reporter induction normalized to cells transfected with the empty expression vector. Error bars indicated the SEM.

NFκB activation in OSU infected cells first was evaluated with the p50 TransAM ELISA, which measures phosphorylated p50 subunit binding to DNA. MA104 cells were infected with A5-16, NCDV, or OSU, and whole cell lysates were harvested six hours post infection (hpi). The amount of activated p50 was significantly higher in A5-16 and NCDV infected cell lysates compared to lysates from mock infected cells (Figure 2A and Figure S1A). By contrast, the amount of activated p50 in OSU infected cell lysates was not different from mock infected controls. The NFκB p50 subunit most commonly forms a p65-p50 heterodimer with transactivation activity [25]. However, p50 also forms homodimers that act as repressors when bound to promoters because the p50 subunit lacks a transactivation domain [32]. The ELISA did not detect elevated levels of p50 capable of binding DNA in OSU infected cells, indicating that infection did not induce formation of the activated p65-p50 heterodimer or the inhibitory p50 homodimer.

Given the previously defined role for NSP1 in IRF degradation, we asked whether NSP1 played a role in modulating the activity of NFκB. 293-TLR3 cells were co-transfected with pO-NSP1 or pN-NSP1, and a luciferase reporter plasmid that contained tandem repeats of κB binding sites. The cells then were treated with polyI:C, and reporter expression was analyzed by luciferase assay (Figure 2B). Expression of OSU NSP1 resulted in ∼20-fold inhibition of NFκB promoter-driven reporter gene expression relative to cells transfected with the empty vector. A five-fold inhibition of luciferase expression was observed in cells expressing NCDV NSP1, indicating some degree of NFκB inhibition by this strain. Previously, we showed that OSU NSP1 accumulates to higher levels than NCDV NSP1 in transfected 293 cells. Immunoblot analysis of cell lysates used for the luciferase reporter experiments showed that NCDV NSP1 and OSU NSP1 were expressed at equal levels (Figure S2), indicating the difference in fold-inhibition between NSP1 of these two strains is not due to differences in levels of expression. Taken together, these data suggested that a predominant mechanism of IFN antagonism in OSU infected cells was interference with activation of NFκB, and that OSU NSP1 alone could mediate this effect. Moderate inhibition of NFκB activation in NCDV infected cells and NCDV NSP1-expressing cells also was observed. However, data from the p50 DNA binding ELISA and reporter gene assay together suggest NCDV is less efficient than OSU in blocking activation of NFκB.

### Localization and activation of NFκB subunit p65

**Figure 3:**
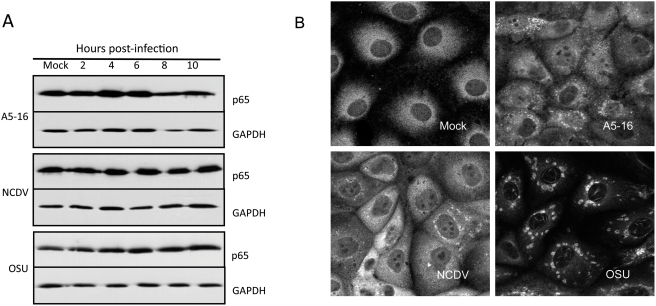
NFκB subunit p65 is stable in rotavirus infected cells. (A) MA104 cells were infected with three pfu/cell of the indicated virus strain. Lysates were prepared at 2, 4, 6, 8, and 10 hpi and the abundance of p65 was determined by immunoblot using anti-p65 antibody. Blots were probed with anti-GAPDH as a loading control. (B) The subcellular localization of p65 in MA104 cells infected with three pfu/cell of the indicated virus strain was determined at six hpi by confocal microscopy (63×, NA 1.40). Cells were stained with anti-p65 antibody, followed by Alexa Fluor 594-conjugated goat anti-rabbit IgG.

The fact that IRF3 is targeted for proteasome degradation by some rotavirus strains led us to test whether degradation of NFκB subunits was responsible for the block to NFκB activation. Whole cell lysates from mock infected and infected cells were collected every two hours for ten hours, and the abundance of p65 (Figure 3A) and p50 (data not shown) was analyzed by immunoblot. The levels of both p65 and p50 in cells infected with the three viruses were equal to the level detected in mock infected cells, indicating that degradation of these subunits was not induced by any of the virus strains tested.

**Figure 4:**
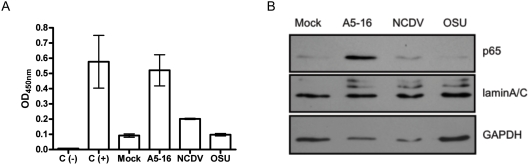
p65 activation and nuclear translocation. MA104 cells were infected with A5-16, OSU or NCDV at an moi of three pfu/cell. Six hours post-infection, nuclear and cytoplasmic fractions were separated with the nuclear extract kit following the manufacturer's instructions (Active Motif). (A) p65 activation measured by p65 TransAm ELISA. Error bars are the standard error of the mean. (B) Nuclear fractions were probed with anti-p65 antibody, anti-laminA/C (nuclear, BD Biosciences), and anti- GAPDH (cytoplasmic) antibodies.

Inhibition of NFκB reporter activation and stability of NFκB subunits in OSU and NCDV infected cells led us to test whether p65 was transcriptionally functional. Cells were mock infected or infected as before, and activated p65 in nuclear fractions was measured with the p65 TransAM ELISA. Consistent with the reporter assays, the amount of activated p65 in OSU and NCDV infected cells was significantly less than that in A5-16 infected cells (Figure 4A). The amount of nuclear p65 also was measured by immunoblot (Figure 4B). The results indicated that the highest amount of p65 was present in the A5-16 fractions, consistent with induction of NFκB dependent gene expression in A5-16 infected cells. In contrast, very little p65 was detected in fractions from OSU and NCDV infected cells. The cytoplasmic carryover in the fractions of all the samples shown by the presence of GAPDH is nearly identical, including the mock infected control. While these data do not quantitatively assess the amount of p65 that translocates to the nucleus compared to what remains in the cytoplasm, the relative amounts of p65 in each fraction can be compared, and clearly illustrate the significant difference between the amount of p65 present in A5-16 lysates and lysates from OSU and NCDV infected cells.

The lack of p65 activation and the low amounts of p65 in the nucleus of OSU and NCDV infected cells led us to further evaluate the subcellular localization by IF microscopy. MA104 cells were infected for six hours with A5-16, NCDV or OSU, and p65 was localized by staining with an anti-p65 antibody (Figure 3B). p65 was distributed in a diffuse pattern throughout the cytoplasm in mock infected cells as expected. In A5-16 and NCDV infected cells, nuclear staining of p65 was evident, indicating some degree of translocation occurred. p65 localization in OSU infected cells was distinct, as it was detected in the nucleus at very low levels and only in filament-like structures that were inconsistent with the typical distribution pattern displayed by p65 in transcriptionally active complexes. The vast majority of p65 in OSU infected cells was exclusively localized to cytoplasmic perinuclear structures that also were observed, but to a lesser extent in A5-16 and NCDV infected cells. These structures were reminiscent of viroplasms formed in rotavirus infected cells, where genome replication, packaging and particle assembly occur. Double staining with anti-p65 and an antibody to structural protein VP6, and analysis by confocal microscopy showed these two proteins co-localized in infected cells, suggesting p65 that does not translocate to the nucleus is associated with viroplasms (Figure S3A, Protocol S1). Co-immunopreciptation of viral proteins with p65 confirmed this association (Figure S3B). The observed filament structures in the nucleus resembled actin fibers formed in the nucleus of herpesvirus infected cells [33], but their origin was not further investigated in this study.

### IκBα is stable in OSU and NCDV infected cells

**Figure 5:**
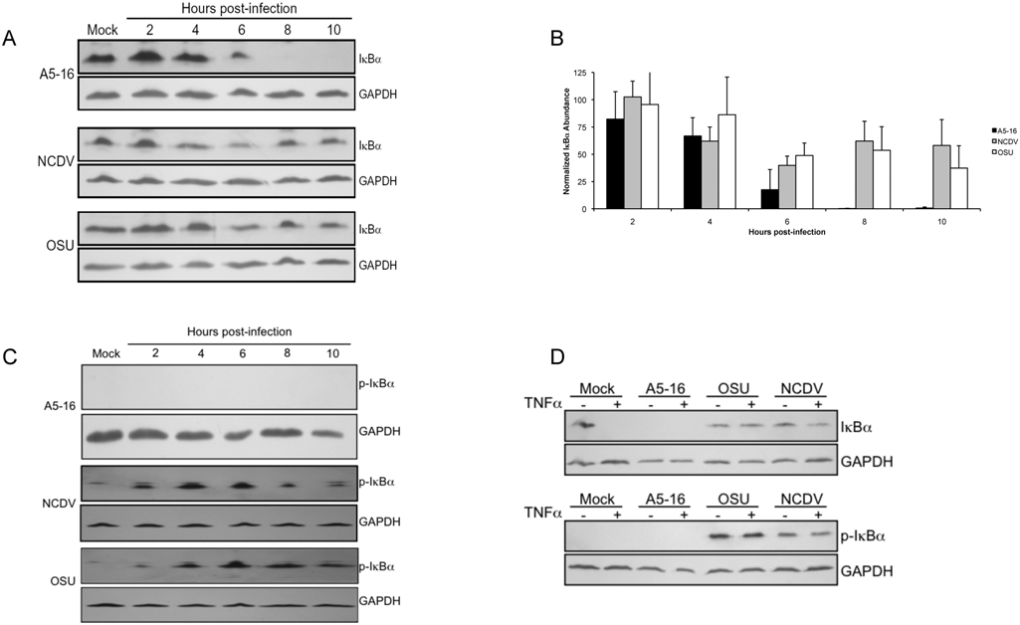
IκBα is stable in cells infected with strains that encode NSP1. (A) MA104 cells were infected with three pfu/cell of the indicated virus strain and lysates were prepared every two hours for ten hours. Immunoblots were probed with anti-IκBα antibody. All blots were probed with anti-GAPDH antibody as a loading control. (B) IκBα levels were quantified by densitometry and are plotted as the ratio of IκBα to GAPDH, normalized to mock infected control. Error bars are the standard error of the mean. (C) Immunblot of lysates in (A) probed with anti-p-IκBα antibody (D) MA104 cells were infected with three pfu/cell of the indicated virus strain. Eight hours post-infection, cells were treated with 50 ng/mL TNFα for 15 minutes. Immunoblots were probed with anti-IκBα or anti-p-IκBα, and anti-GAPDH antibodies. IκBα is not detectable in A5-16 infected cells because it is degraded by six hours post-infection (see panel A).

Phosphorylation of IκBα and subsequent ubiquitination and proteasome degradation is required for activation of NFκB. To assess the activation status of IκBα, lysates from cells infected with each of the three virus strains were prepared every two hours for ten hours, and IκBα was analyzed by immunoblot. IκBα was substantially degraded by six hpi in A5-16 infected cells (Figure 5A), but stable in the presence of MG132, confirming proteasome-dependent degradation (Figure S4). By contrast, IκBα was detected in lysates from NCDV and OSU infected cells through the ten hour time point. Although IκBα was present at each time point through the conclusion of the time course, analysis of band intensities by densitometry from three independent experiments indicated that IκBα was present in NCDV and OSU infected lysates at levels approximately 50% of the mock infected controls (Figure 5B). Therefore, some IκB degradation does occur, most likely prior to accumulation of levels of viral protein sufficient to block this activity.

IκBα is phosphorylated by the IKK complex on serine residues 32 and 36, resulting in recognition and ubiquitination by the SCF^β−TrCP^ E3 ubiquitin ligase complex [18],[34]. To distinguish between a block to phosphorylation of IκBα and a block to subsequent proteasome degradation, immunoblots were probed with an antibody that recognizes the phosphorylated form of IκBα (p-IκBα, Figure 5C). Phosphorylated IκBα was not detected at any time during the infection with A5-16 because this form is rapidly degraded. In OSU and NCDV infected cells, p-IκBα accumulated through ten hpi, with peak accumulation observed at 4–6 hpi. The presence of p-IκBα indicated that activity of the IKK complex was not disrupted by either of these viruses, and that proteasome-mediated degradation of IκBα is inhibited by both.

Degradation of IκBα is rapidly induced in cells treated with TNFα. To determine if IκBα was stable in cells when the stimulus was not virus infection, and to confirm that p-IκBα was stable, we tested whether TNFα induced IκBα degradation in infected cells. MA104 cells were mock infected or infected for eight hours, and then treated with TNFα for 15 minutes. Cell lysates then were probed with antibody to total IκBα or to p-IκBα. The results revealed that upon treatment with TNFα, IκBα was phosphorylated and stable in NCDV and OSU infected cells, but not in mock infected cells (Figure 5D). These data show that treatment with a strong inducer of NFκB does not overcome the virus infection-associated block in this signaling pathway, and further support the conclusion that IκBα degradation is actively inhibited in OSU and NCDV infected cells.

### F-box protein β-TrCP is degraded in OSU and NCDV infected cells

The SCF^β-TrCP^ E3 ligase complex mediates proteasome-dependent degradation of several proteins with roles in regulating cell proliferation, including IκBα, NFκB subunits p100 and p105, cyclin dependent kinases, and β-catenin, among others [35]. SCF^β-TrCP^ is a negative regulator of the Wnt/β catenin signaling pathway, and as such, expression of dominant negative mutants of β-TrCP results in accumulation of β-catenin [36]. We reasoned that if SCF^β-TrCP^ was nonfunctional in infected cells, β-catenin, in addition to IκBα, would accumulate to a level higher than that in mock infected controls. The amount of β-catenin in OSU infected cells at ten hours post infection was consistently higher than mock infected cells, suggesting activity of SCF^β-TrCP^ was impaired (Figure S5).

**Figure 6:**
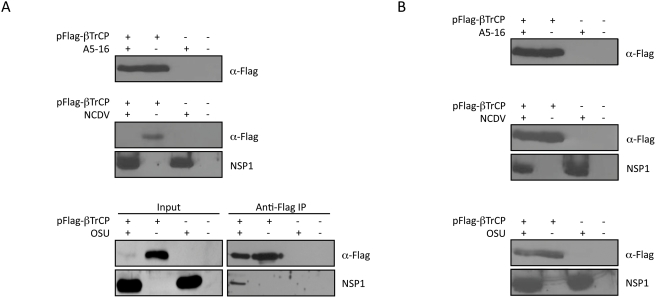
Rotavirus strains that express NSP1 induce proteasome-mediated degradation of β-TrCP. (A) 293 cells were transfected with Flag-tagged β-TrCP (pFlag-βTrCP) and then infected with three pfu/cell of the indicated virus strain. Cell lysates were prepared eight hpi, and immunoblots were probed with anti-Flag antibody (top panel); anti-Flag and anti-NCDV NSP1 antibody (middle panel); or anti-Flag and anti-OSU NSP1 (bottom panel, Input). In the bottom right panel, the lysate from β-TrCP expressing, OSU infected cells was subjected to immunoprecipitation with anti-Flag M2 Agarose Affinity Resin, and blots were probed with anti-Flag antibody or anti-OSU NSP1 antibody (B) The same experiment as described in (A), except that proteasome inhibitor MG132 was added at the time of virus infection to final concentration of 10 µM.

To test the theory that SCF^β-TrCP^ may not be functional in OSU and NCDV infected cells, 293 cells were transfected with a plasmid encoding a Flag-tagged β-TrCP, and then infected with the three strains as in prior experiments. Immunoblots were probed with anti-Flag antibody to detect β-TrCP, or NSP1 polyclonal antibody. In A5-16 infected cells, β-TrCP was present in approximately the same amounts as the transfected, uninfected control (Figure 6A). β-TrCP was not detected in either NCDV infected cells or OSU (input panel) infected cells. These data led us to suspect that β-TrCP may be targeted to the proteasome in cells infected with these two strains. To address this question, cells were transfected with Flag-β-TrCP, and then infected in the presence of proteasome inhibitor MG132. Immunoblots were probed with anti-Flag or anti-NSP1 antibodies. The results showed that when proteasome activity is inhibited, β-TrCP is stable in infected cells (Figure 6B). The fact that β-TrCP was stable in cells infected with A5-16 in the absence of MG132, suggested that NSP1 played a role in this process.

**Figure 7:**
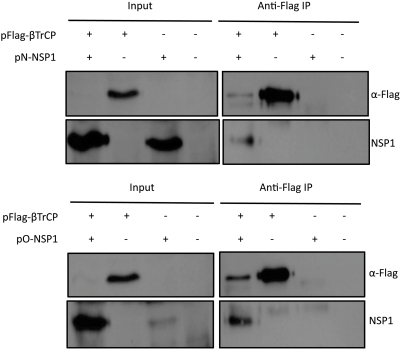
NSP1 expressed in isolation binds to and induces degradation of β-TrCP. 293 cells were co-transfected with pFlag-β-TrCP and either pN-NSP1 or pO-NSP1. Forty hours post-transfection, MG132 was added to the media to a final concentration of 10 µM and the cells were incubated an additional eight hours. 10% of each sample was set aside as input and the remaining lysate was subjected to immunoprecipitation with anti-FLAG M2 Agarose Affinity Resin (Sigma). Immunoblots were probed with anti-Flag and anti-myc (NSP1 detection) antibodies.

NSP1-directed degradation of IRF3 requires an interaction between the two proteins. We tested whether NSP1 interacted with β-TrCP. 293 cells were co-transfected with plasmids encoding NSP1 and Flag-β-TrCP in the presence of MG132, and co-immunoprecipitations were performed with anti-Flag antibody, followed by immunoblot with either anti-Flag or anti-myc antibody to detect NSP1. NSP1 of both OSU and NCDV co-immunoprecipitated with β-TrCP when cells were treated with MG132 to protect β-TrCP from degradation (Figure 7). We note that β-TrCP is not detected in the input lanes even in the presence of MG132, in contrast to the data shown in Figure 6B. We believe this is a consequence of the experimental conditions, in that an eight hour treatment with MG132 is insufficient to allow β-TrCP to accumulate to detectable levels when it is co-expressed with NSP1 for 40 hours prior to addition of the proteasome inhibitor. The immunoprecipitation reactions in this case effectively concentrate the amount of β-TrCP remaining so that it is detected in complex with NSP1. In addition, there is an obvious difference in the amount of OSU NSP1 present in cells that were co-transfected with β-TrCP compared to those transfected with the NSP1 plasmid alone (Figure 7, pO-NSP1 input lanes), such that NSP1 appears stabilized in the presence of β-TrCP. We have observed an increase in the levels of expression of both NSP1 and EYFP when this construct is co-transfected with the β-TrCP plasmid. The reasons for the increase are not known, but as shown by the data in Figure 6, the presence of β-TrCP has no effect on the levels of NSP1 synthesized in native virus infected cells. The interaction between NSP1 and β-TrCP was confirmed to occur in OSU infected cells by co-immunoprecipitation of NSP1 with β-TrCP from Flag-β-TrCP expressing cells (see Figure 6A, IP panel).

## Discussion

It is clear that viruses encode proteins that down-regulate cellular immune responses, yet the strategies are disparate, and include interference with both innate and adaptive pathways. We report a novel immune evasion mechanism in which a viral nonstructural protein targets the substrate recognition component of a multisubunit cellular E3 ligase for proteasome degradation. At least one functional consequence of β-TrCP degradation relevant to induction of the innate immune response is inhibition of NFκB activation and IFNα/β expression. However, SCF^β-TrCP^ also regulates stability of proteins involved in control of cell proliferation including β-catenin, Emi1, and cyclin dependent kinases, among many others [35]. As such, over-expression of β-TrCP and consequent constitutive activation of NFκB has been reported in several tumor cell lines [37],[38],[39],[40]. It stands to reason then, that in the context of viral infection, the effects of the absence of β-TrCP on the proliferative state of the cell also may play a role in modulating virus replication. Blocking NFκB activation in response to cell stress will result in amplification of a pro-apoptotic response. Several studies have shown that rotavirus infection induces apoptosis both in vitro and in vivo, and mechanisms including most recently, induction by the intrinsic pathway, have been reported [41],[42],[43]. Together, the data suggest that inducing apoptosis may be advantageous for rotavirus replication and spread, and it remains to be determined whether blocking apoptosis will affect virus spread or progeny production.

While targeted degradation of β-TrCP by NSP1 is unique, viral manipulation of the ubiquitin-proteasome system is not [25]. Some viral proteins modulate the ubiquitin-proteasome system by redirecting cellular E3 ligases to alternative substrates or directly interfering with their function. For example, V proteins of some viruses in the *Paramyxoviridae* target STAT1 and/or STAT2 for proteasome degradation, thus preventing assembly of ISGF3 [44],[45],[46]. IRF7 is required for a maximal IFN response and is a substrate for the Kaposi's sarcoma herpesvirus (KSHV) RTA ligase [47]. Herpes simplex virus protein ICP0 has been well characterized, and induces proteasome-dependent degradation of the stress-related kinase DNA-PK [48] and promyelocytic leukemia protein [49],[50]. Viral infectivity factor (VIF) encoded in the HIV genome forms a ligase complex through interactions with the RING domain-containing protein Rbx1/Roc1, and recruits APOBEC3G as a substrate for ubiquitination, thus functioning as an F-box protein surrogate in cellular complexes [51].

Another HIV protein that manipulates the ubiquitin-dependent proteasome system and is particularly relevant to the work reported here is Vpu, most noted for its role in directing degradation of CD4 [52]. Vpu binds CD4 and β-TrCP, resulting in polyubiquitination of CD4 and proteasome degradation, but Vpu itself is not degraded through this interaction [52]. It has been suggested that Vpu blocks NFκB activation in infected cells by acting as a competitive inhibitor for β-TrCP binding, and consequently stabilizes IκB [52]. NSP1-dependent proteasome-mediated degradation of β-TrCP reveals a new mechanism for interfering with the function of this protein. It is now evident that major players in immune response signaling networks are targeted by viruses in several families, and therefore, consideration of β-TrCP as another important target of IFN antagonism by multiple viruses seems warranted.

The conserved N-terminal cysteine-histidine rich region of NSP1 does not conform to standard RING domain signatures. However, mutational analysis has shown that cysteine and histidine residues in this region are required to direct IRF3 degradation in transfected cells [14]. Direct evidence of intrinsic E3 ligase activity of NSP1 has not yet been reported, and in this context, it is appropriate to consider the mechanism by which NSP1 “hijacks” the cellular ubiquitination machinery and directs proteasome degradation of selected substrates. RING domain-containing E3 ligases are either unimolecular or multisubunit, and viral E3 ligases exist in both categories [25]. Unimolecular E3s recruit the cognate E2 through a RING domain, and the substrate through protein-protein interactions in a separate domain of the molecule. Single subunit E3s include cellular proteins c-Cbl [53], Mdm2 [54], and XIAP [55], and viral proteins RTA [47]and ICP0 [56], all of which are relatively large proteins of >450 amino acids. In multisubunit ligases such as SCF^β-TrCP^, the RING domain protein Rbx1/Roc1 is ∼100 amino acids and the substrate recognition component is a separate protein in the complex. NSP1 could assemble in SCF complexes, thus functioning as the substrate recognition component and RING finger protein of a multisubunit E3. The molecular weight of NSP1 (∼490 amino acids) and predicted structure is most consistent with single subunit E3s, and we recently reported that NSP1 regulates its own stability in a proteasome-dependent manner [14]. This auto-regulatory capability is characteristic of unimolecular cellular and viral E3s that have experimentally defined intrinsic ligase activity [53],[54]. A refined definition of the mechanism by which NSP1 functions in the ubiquitin-dependent proteasome pathway requires study of additional cellular interaction partners, including those specifically associated with ubiquitin ligase complexes. Likewise, the motifs in cellular proteins that dictate their recognition as NSP1 substrates are not known, and determining the range of activity of NSP1 will be important to decipher.

Since we had observed that IRF3 was activated in OSU infected cells but IFNβ transcription still was inhibited, we expected an alternative mechanism was operational and independent of IRF3. The discovery that β-TrCP was degraded in NCDV infected cells as well was unexpected, but not wholly surprising, because redundancy in mechanisms of viral interference with IFN signaling is well documented. NFκB activation was inhibited in NCDV infected cells, but the magnitude was much less than that observed in OSU infections. Therefore, IRF3 degradation in NCDV infected cells may be the dominant and most efficient mechanism of NSP1-mediated IFN antagonism for this strain, and perhaps other strains known to target IRF3. In contrast, OSU NSP1 does not strongly bind IRF3 in these cells lines, and inhibition of NFκB activity by degradation of β-TrCP is dominant. Substrate specificity of NSP1 thus may define the prominent mechanism of IFN antagonism in different rotavirus strains.

The results of these studies also suggest rotaviruses utilize more than one mechanism to down-regulate NFκB-dependent gene expression that is independent of NSP1. There is similarity in the localization of p65 to viroplasms in cells infected with each virus, including A5-16 which does not encode NSP1, the obvious difference being that nuclear accumulation of transcriptionally active p65 in OSU infected cells was nearly completely blocked. This observation suggests OSU is more efficient at sequestering p65 in the cytoplasm and by extension, inhibiting NFκB activation, than NCDV and A5-16. Sequestration of an NFκB subunit in viroplasms and interactions with viral structural proteins is a novel observation. We have shown that p65 co-immunoprecipitates with at least three rotavirus proteins, and it will be of interest to determine the role of these proteins in the altered distribution of p65. It is possible that the degree of p65 sequestration depends on strain specific differences in kinetics or efficiency of viroplasm formation in infected cells.

NFκB-dependent cytokine induction and microarray data have shown increased expression of NFκB-regulated genes during rotavirus infection [57],[58]. Our data generally are not in conflict with these results. We observe moderate degradation of IκBα in both OSU and NCDV infected cells at early times post-infection, and it is plausible that some NFκB-dependent gene expression is induced. This makes sense given the time-lapse between activation of signal transduction pathways and accumulation of NSP1 in the cell. In this context, viral interference with these pathways functions to down-regulate the response instead of blocking its initial induction. Other rotaviruses reported so far to activate NFκB include among others, simian and rhesus strains that target IRF3 for degradation. Although we have not determined whether NFκB is activated in cells infected with these strains, we predict activity similar to NCDV. Continued analysis of the ways multiple rotavirus strains modulate the NFκB pathway will test this prediction.

Use of viruses that are unable to block IFN responses is becoming an attractive approach to development of attenuated virus vaccines [1],[59],[60],[61],[62],[63]. Generation of rotavirus vaccines containing strains with mutations within NSP1 may result in additional vaccine formulations that consist of attenuated virus isolated from homologous hosts. In this context, careful analysis of the functions of NSP1 in regulation of innate immune responses will be important for future studies.

## Materials and Methods

### Cells, viruses, and NSP1 expression plasmids

MA104 cells were grown in M199 media (Mediatech) supplemented with 5% FBS (Atlanta Biologicals). HEK293 cells were grown in RPMI 1640 media (Mediatech) supplemented with 10% FBS, 10 mM HEPES, 1× nonessential amino acids, 2 mM L-glutamine, and 1 mM sodium pyruvate. 293-TLR3 cells (kindly provided by Dr G. Sen, Cleveland Clinic, Cleveland, OH), were grown in the same conditions as 293 cells, except that the media was supplemented with 400 µg/mL of G418.

Isolation and characterization of rotavirus strains A5-16, NCDV, and OSU has been described [28],[64],[65],[66]. To prepare virus stocks, MA104 cells were infected at a multiplicity of infection (MOI) of 0.05, and harvested when ∼95% cytopathic effect was observed. Culture media was clarified of cell debris by centrifugation at 2000×*g* for 10 minutes, and the virus in the supernatant was concentrated by centrifugation at 26,000 rpm in an SW28 rotor (Beckman) for two hours at 4°C. Virus pellets were suspended in serum-free M199, and stored in aliquots at −80°C. Virus titers were determined by standard plaque assay.

Construction of expression plasmids encoding OSU NSP1 (pO-NSP1) and NCDV NSP1 (pN-NSP1) has been described [14]. A myc epitope tag is fused to the NSP1 coding sequence of both for detection by immunoblot.

### RT-PCR

MA104 cells were infected with trypsin-activated virus at an MOI of three pfu/cell in serum-free M199. Eight hours post-infection (hpi), total cell RNA was extracted using TRIzol reagent (Invitrogen). RNA was treated with ten units of DNAse for 30 minutes at 37°C, and then extracted with phenol/chloroform. Reverse transcription reactions were performed with random hexamers (Promega) and Superscript II reverse transcriptase (Invitrogen), incubated for 50 minutes at 42°C. PCR reactions were performed with Taq polymerase (Promega). Gene-specific primers included: IFNβ (forward 5′ - CTC CTC CAA ATT GCT CTC CTG – 3′; reverse 5′ – GCA AAC TGC TCA CGA ATT TTC C – 3′), ISG56 (forward 5′ – AAC ACC TGA AAG GCC AGA ATG AGG-3′; reverse 5′ – AAG ACA GAA GTG GGT GTT TCC TGC-3′), IP-10 (forward 5′ – CTG CGA TTC TGA TTT GCT GCC – 3′; reverse 5′ – GGA GAT CTT TTA GAC ATT TCC TTG CTA ACT GC – 3′), and β-actin (forward 5′ – CAT GTT TGA GAC CTT CAA CAC – 3′; reverse 5′ – CAT CTC CTG CTC GAA GTC TAG – 3′).

### Reporter gene assays

293-TLR3 cells were transfected using TransIT-293 Transfection Reagent (Mirus) according to the manufacturer's instructions. The luciferase control plasmid phRL-CMV was purchased from Promega. IFNβ-Luc was kindly provided by Dr J. Hiscott, McGill University, Montreal, Quebec, CA, and pNFκB-Luc *Cis* Reporter Plasmid was purchased from Stratagene. Forty hours post-transfection, polyI:C (GE Healthcare) was added to the media at final concentration of 100 µg/mL. Cell lysates were harvested eight hours post-treatment and reporter activity was measured using the Dual Luciferase Assay (Promega) and a Lumat LB 9507 luminometer (EG&G Berthold). Each transfection was performed in triplicate in two independent experiments.

### NFκB activation by TransAm ELISA

NFκB subunit p50 was measured by the p50 TransAM ELISA (Active Motif). Whole cell extracts were prepared six hpi in Lysis Buffer AM1. Protein concentration was determined with the RC DC Protein Assay (Bio-Rad Laboratories) and 20 µg of extract were used in a TransAM p50 Activation Assay (Active Motif). Specificity controls provided in both the p50 and p65 assays are shown in Figure S1. Three independent experiments were performed and *p*-values were determined by Student's unpaired *t*-test.

Nuclear p65 was analyzed with the p65 TransAM ELISA. Nuclear and cytoplasmic fractions were prepared six hpi with the nuclear extract kit following the manufacturer's instructions (Active Motif). Protein concentration was determined with the RC DC protein assay and five µg of nuclear extract were used in the assay (Active Motif). Three independent experiments were performed and *p*-values were determined by Student's unpaired *t*-test.

### Immunoblots

Cell lysates were harvested at the indicated times post-infection in RIPA buffer (150 mM NaCl, 1% sodium deoxycholate, 1% Triton X-100, 0.1% SDS, 10 mM Tris-HCl pH 7.2). Immunoblots were performed as previously described [23]. Primary antibodies against the following proteins were used: p65 (Rockland), IκBα (Cell Signaling), IκBα phosphorylated on serine 32 (Cell Signaling), GAPDH (Ambion), anti-FLAG (Sigma), anti-NCDV NSP1 and anti-OSU NSP1. Rabbit polyclonal antiserum to OSU NSP1 generated against a synthetic peptide near the C terminus of the protein and was made by Open Bioscience, Inc.

### Immunoprecipitations

For immunoprecipitation from infected cell lysates, HEK293 cells were mock transfected or transfected with pCMV-Flag-β-TrCP2 (kindly provided by Dr. W. Harper, Department of Pathology, Harvard Medical School, Boston, MA). Twenty hours post-transfection, the media was changed to serum-free RPMI 1640 and the cells were infected with three pfu/cell of OSU or NCDV. Cells were harvested eight hpi in 500 µL FLAG immunoprecipitation lysis buffer (50 mM Tris-HCl, pH 7.5, 150 mM NaCl, 1% Triton X-100, 1 mM EDTA) supplemented with protease inhibitor cocktail (Roche Diagnostics). Lysates were passed through a 30 gauge needle five times and subsequently pre-cleared by centrifugation for 10 minutes at 10,000×g. Ten percent of each sample was set aside as input, and the FLAG-tagged β−TrCP was immunoprecipitated from the remaining lysate using anti-FLAG M2-Agarose Affinity Gel (Sigma) according to the manufacturer's recommendations. The input and eluted samples were used in immunoblot analysis using antibodies against FLAG (Sigma), and OSU NSP1 or NCDV NSP1. Immunoprecipitations from cells co-transfected with pFlag-β-TrCP and pN- or pO-NSP1 were performed the same way, except that MG132 was added to the media 40 hours post-transfection, followed by an additional eight hour incubation.

### Immunofluorescence microscopy

MA104 cells were grown in four-well chamber slides (BD Falcon) and infected with three pfu/cell using rotavirus stocks that were activated with 10 µg/mL Worthington trypsin for 30 minutes at 37°C. Six hpi, the cells were fixed with paraformaldehyde (2% w/v in PBS) for 10 minutes at room temperature and then permeabilized with methanol for 10 minutes at −20°C. The samples were incubated in blocking solution (PBS supplemented with 5% v/v horse serum and 5% v/v goat serum) overnight at 4°C. Primary antibody diluted in blocking serum was added and cells were incubated for one hour at room temperature, followed by three rinses with PBS. The primary antibodies were mouse anti-IRF3 (Santa Cruz Biotechnology) or rabbit anti-p65 (Rockland). The cells were then stained the appropriate Alexa Fluor-conjugated goat secondary antibodies (Invitrogen) and mounted in ProLong Gold (Invitrogen) under a glass cover slip. Cells stained for IRF3 were viewed on an AxioImager.A1 microscope (Zeiss) using a 40× (0.75 NA) objective lens. The images were obtained using an AxioCam MRc5 camera (Zeiss) and analyzed with the Axiovision version 4.6.3 (Zeiss) software package. Exposure parameters were equal for images of each sample. Cells stained for p65 were viewed on an LSM 510 Meta confocal microscope (Zeiss) using a 63× (1.40 NA) objective lens. Excitation of Alexa Fluor 594-conjugated secondary antibody was achieved using a 543 nm helium-neon laser. The pinhole was set to 1 Airy unit. Images were obtained with each line being scanned eight times to increase the signal-to-noise ratio. The images were analyzed with the LSM Image Browser (Zeiss).

## Supporting Information

Figure S1 Specificity of p50 and p65 binding in TransAM assays. MA104 cells were infected with A5-16, NCDV, or OSU as described in the text. Lysates were prepared six hours post infection and subjected to the (A) p50 or (B) p65 binding assay. Competitor oligonucleotides are wildtype (w) or mutant (m).
(3.00 MB TIF)

Figure S2 Immunoblot analysis of lysates used in luciferase reporter assays. Cell lysates used in the NFκB and IFNβ reporter luciferase assays (Figure 2B and Figure 1C, respectively) were analyzed by immunoblot with anti-myc antibody to detect myc-tagged NSP1. The asterisk indicates a protein that cross-reacts with the myc antibody. The blots were probed for GAPDH as a loading control.
(0.37 MB TIF)

Figure S3 p65 co-localizes with VP6 and NSP2 in viroplasms. (A) The cellular localization of p65, VP6, and NSP2 in MA104 cells infected with three pfu/cell of the indicated virus strain was determined by confocal microscopy (63×, NA 1.40) at six hpi. The zoom function was used to allow for analysis of individual cells. (B) Interactions between p65 and rotavirus proteins were determined by co-immunoprecipitation. Rotavirus proteins were radiolabeled for six hours in MA104 cells infected with ten pfu/cell of the indicated virus strain (details in Materials and Methods). Cell lysates were prepared and 10% of each sample was set aside as input control. The remaining lysate was divided equally for immunoprecipitation with anti-p65 antibody. Input controls and immunoprecipitated samples were separated by SDS-PAGE, and radiolabeled proteins were detected by autoradiography.
(2.16 MB TIF)

Figure S4 IκBα degradation is proteasome dependent. MA104 cells were infected with A5-16 in the absence or presence of 10 µM MG132. Immunoblots were probed with anti-IκBα and anti-GAPDH.
(3.00 MB TIF)

Figure S5 β-catenin is stabilized in OSU infected cells. Cell lysates were collected ten hpi from 293 cells infected with three pfu/cell of the indicated virus strain. The abundance of β-catenin was determined by immunoblot and densitometry normalized to GAPDH. The level of β-catenin in OSU infected cells was ∼2–4 fold higher than in mock infected or A5-16 infected cells.
(0.72 MB TIF)

Protocol S1 Supplemental methods
(0.04 MB DOC)
