# Supplementary figures and images for: Rotavirus NSP1 Inhibits NFκB Activation by Inducing Proteasome-Dependent Degradation of β-TrCP: A Novel Mechanism of IFN Antagonism
Source: PLoS Pathog. 2009 Jan 30;5(1):e1000280. doi: 10.1371/journal.ppat.1000280 (PMC2627925; doi:10.1371/journal.ppat.1000280)

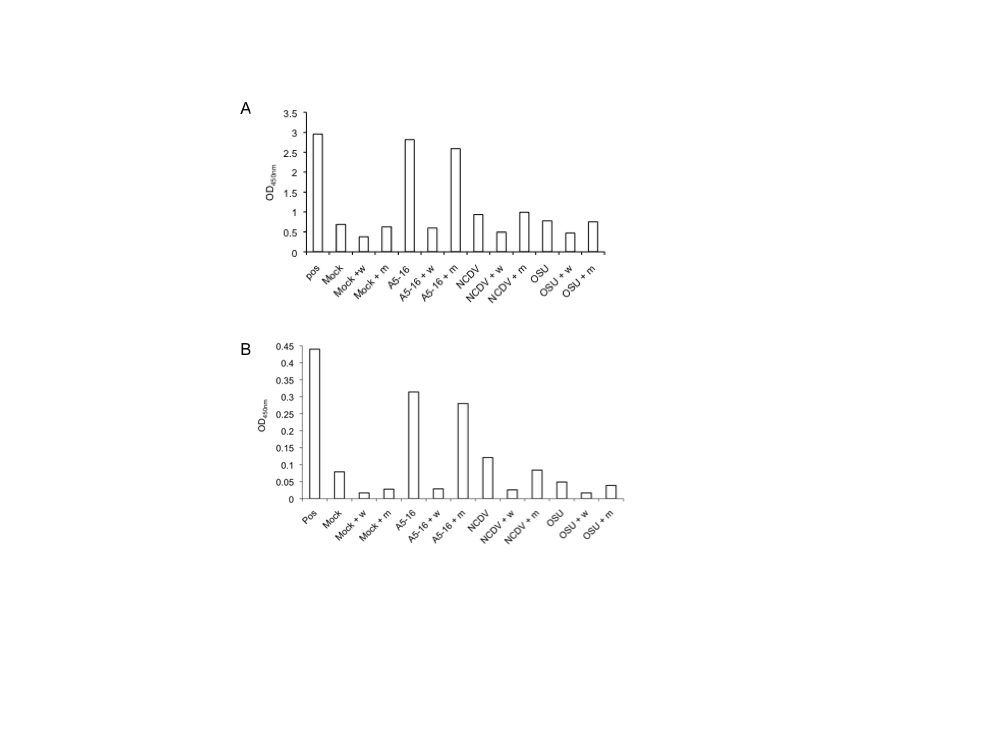

Supplement: Figure S1 — Specificity of p50 and p65 binding in TransAM assays. MA104 cells were infected with A5-16, NCDV, or OSU as described in the text. Lysates were prepared six hours post infection and subjected to the (A) p50 or (B) p65 binding assay. Competitor oligonucleotides are wildtype (w) or mutant (m). (3.00 MB TIF) [file ppat.1000280.s001.tif]

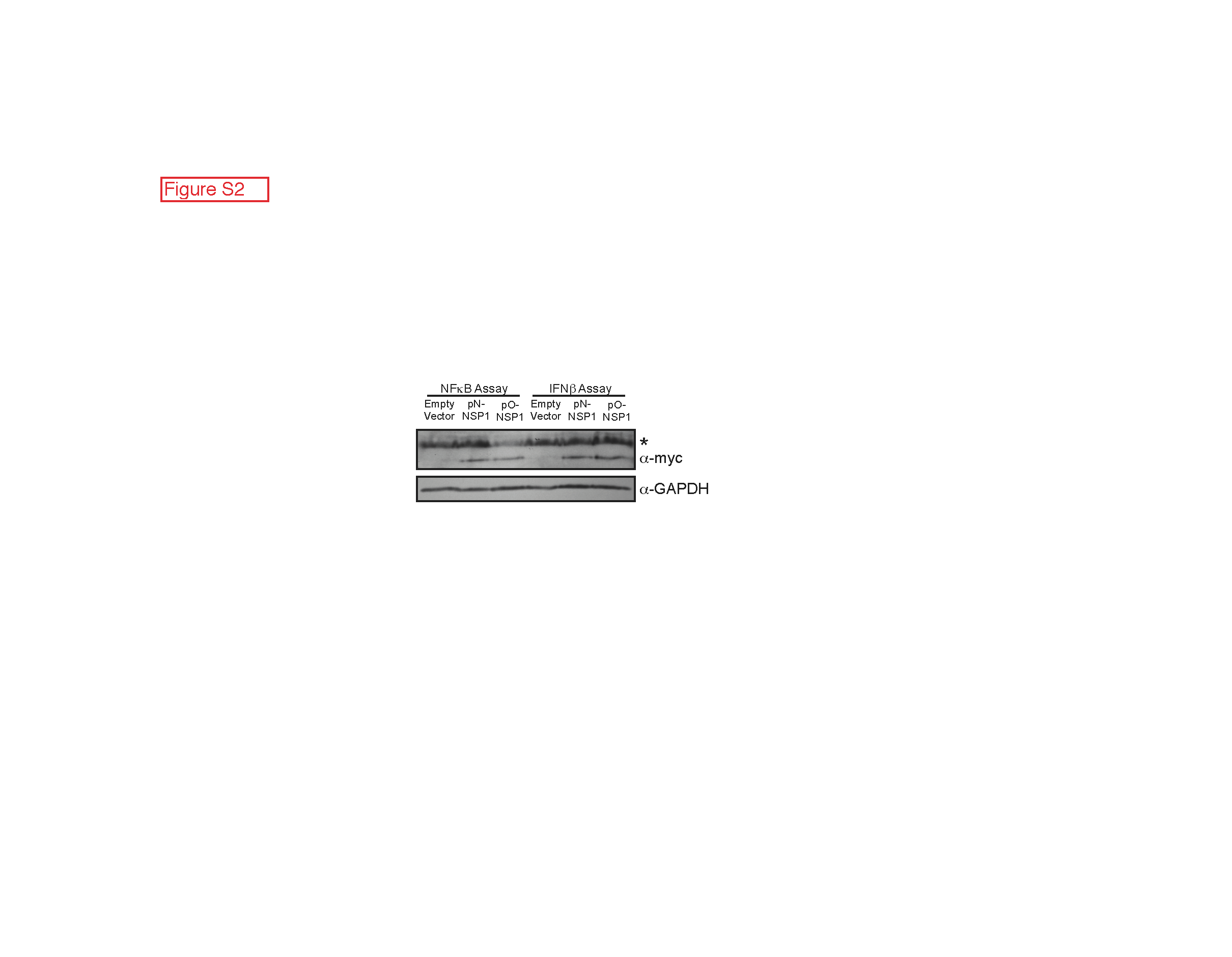

Supplement: Figure S2 — Immunoblot analysis of lysates used in luciferase reporter assays. Cell lysates used in the NFκB and IFNβ reporter luciferase assays (Figure 2B and Figure 1C, respectively) were analyzed by immunoblot with anti-myc antibody to detect myc-tagged NSP1. The asterisk indicates a protein that cross-reacts with the myc antibody. The blots were probed for GAPDH as a loading control. (0.37 MB TIF) [file ppat.1000280.s002.tif]

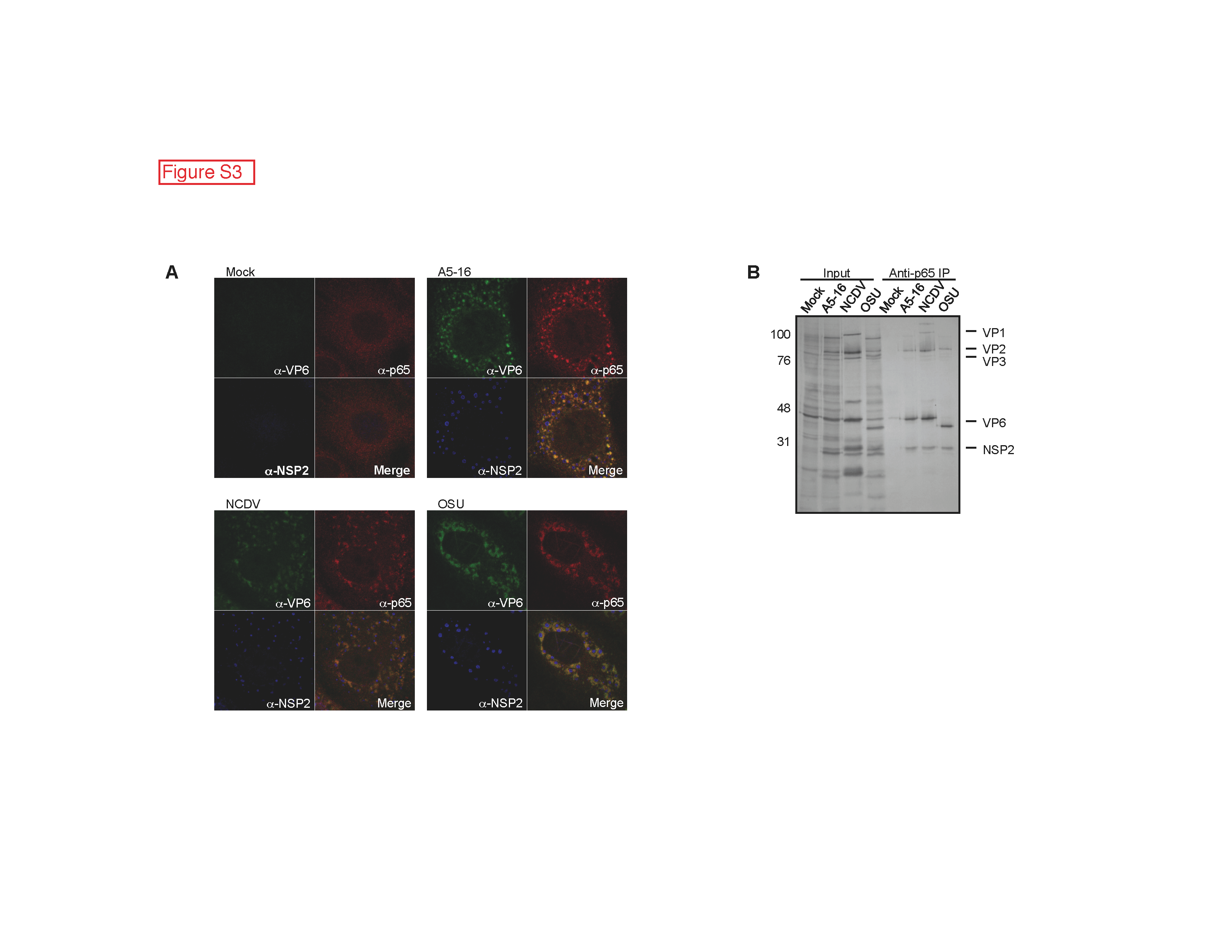

Supplement: Figure S3 — p65 co-localizes with VP6 and NSP2 in viroplasms. (A) The cellular localization of p65, VP6, and NSP2 in MA104 cells infected with three pfu/cell of the indicated virus strain was determined by confocal microscopy (63×, NA 1.40) at six hpi. The zoom function was used to allow for analysis of individual cells. (B) Interactions between p65 and rotavirus proteins were determined by co-immunoprecipitation. Rotavirus proteins were radiolabeled for six hours in MA104 cells infected with ten pfu/cell of the indicated virus strain (details in Materials and Methods). Cell lysates were prepared and 10% of each sample was set aside as input control. The remaining lysate was divided equally for immunoprecipitation with anti-p65 antibody. Input controls and immunoprecipitated samples were separated by SDS-PAGE, and radiolabeled proteins were detected by autoradiography. (2.16 MB TIF) [file ppat.1000280.s003.tif]

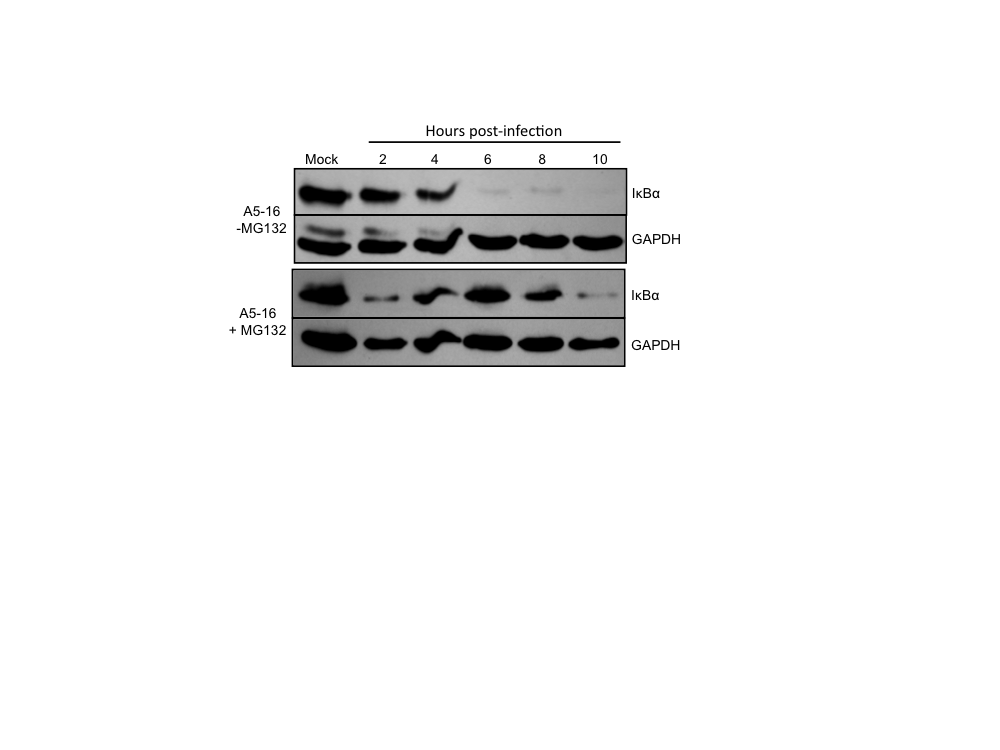

Supplement: Figure S4 — IκBα degradation is proteasome dependent. MA104 cells were infected with A5-16 in the absence or presence of 10 µM MG132. Immunoblots were probed with anti-IκBα and anti-GAPDH. (3.00 MB TIF) [file ppat.1000280.s004.tif]

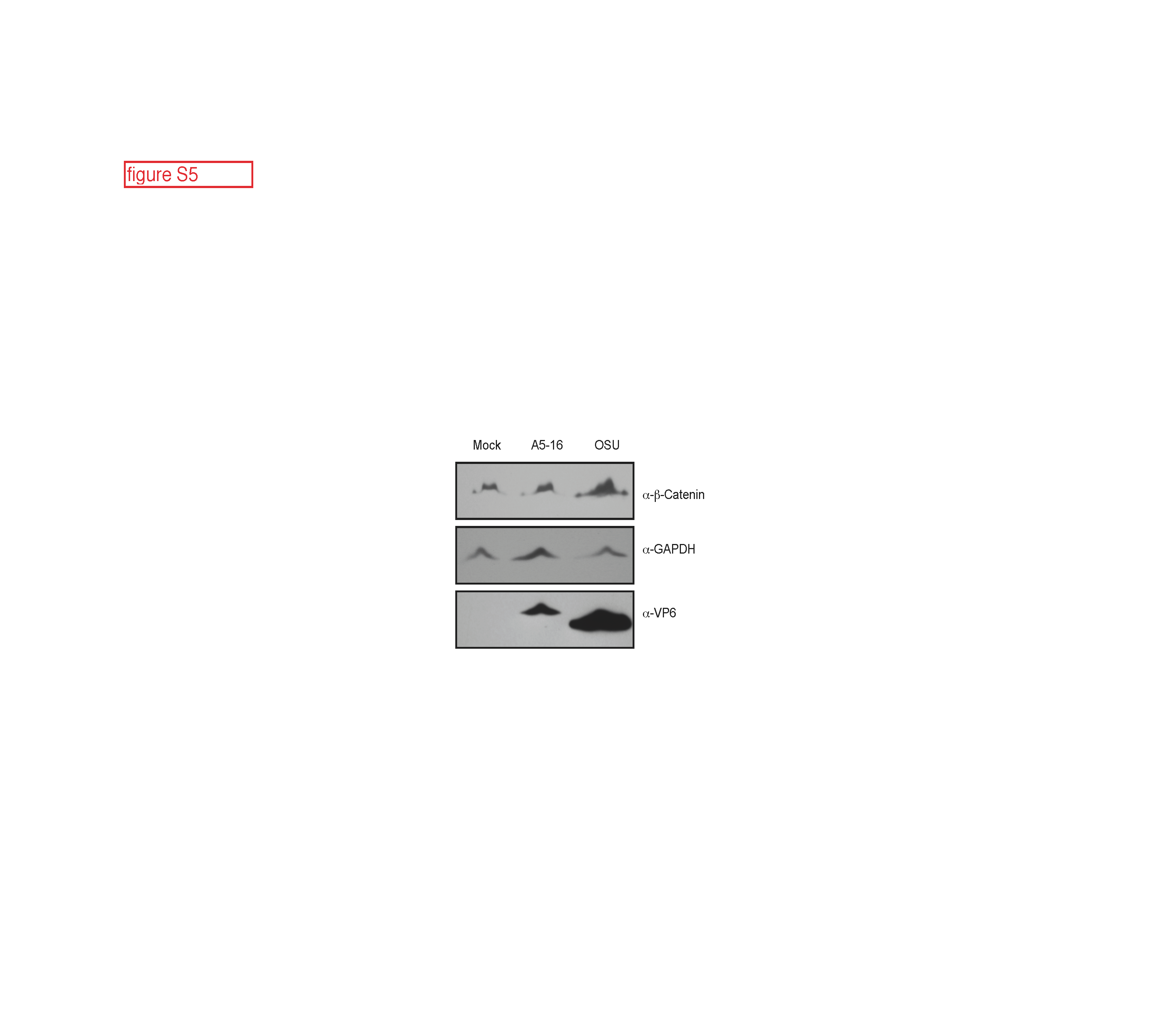

Supplement: Figure S5 — β-catenin is stabilized in OSU infected cells. Cell lysates were collected ten hpi from 293 cells infected with three pfu/cell of the indicated virus strain. The abundance of β-catenin was determined by immunoblot and densitometry normalized to GAPDH. The level of β-catenin in OSU infected cells was ∼2–4 fold higher than in mock infected or A5-16 infected cells. (0.72 MB TIF) [file ppat.1000280.s005.tif]
