# Supplementary material for: Rotavirus NSP1 Inhibits NFκB Activation by Inducing Proteasome-Dependent Degradation of β-TrCP: A Novel Mechanism of IFN Antagonism
Source: PLoS Pathog. 2009 Jan 30;5(1):e1000280. doi: 10.1371/journal.ppat.1000280 (PMC2627925; doi:10.1371/journal.ppat.1000280)
Supplement: Protocol S1 — Supplemental methods (0.04 MB DOC) [file ppat.1000280.s006.doc]

**Supplemental Methods**

**Competitive inhibitor controls for TransAM assays (Figure S1)** TransAM p50 and p65 assays were performed according to the manufacturer’s protocol. Conditions of infection and lysate harvest are as described in Materials and Methods.

**NSP1 levels in reporter gene assays (Figure S2)** Equivalent volumes of cell lysates used in the NFκB and IFNβ dual luciferase assays (Figures 2B and 1C, respectively) were probed in immunoblots with anti-c-myc antibody (Clontech) to detect NSP1 and anti-GAPDH (Ambion).

**Confocal microscopy (Figure S3A)** Samples were prepared as described in Materials and Methods. The primary antibodies used were rabbit anti-p65 (Rockland), mouse anti-VP6, and guinea pig anti-NSP2 (kindly provided by Dr M. Estes, Baylor College of Medicine, Houston, TX). VP6, NSP2, and p65 triple stained cells were viewed on an LSM 510 Meta confocal microscope (Zeiss) using a 63X (1.40 NA) objective lens. The zoom function was used to obtain the image of a single cell. Excitation of Alexa Fluor 488, Alexa Fluor 594, and Alexa Fluor 633 was achieved using a 488 nm argon laser, a helium-neon 543 nm laser, and a helium-neon 633 nm laser, respectively. The pinhole was set to 1 Airy unit for each channel. Images were obtained using the multitrack scanning mode to minimize crosstalk between channels and each line was scanned eight times and averaged to increase the signal-to-noise ratio. The images were analyzed with the LSM Image Browser (Zeiss).

**Co-immunoprecipitations (Figure S3B)** MA104 cells were grown to 90% confluency in M199 supplemented with 5% FBS. Growth medium was changed to M199 supplemented with 2% FBS and 5 μg/mL actinomycin D, and cells were incubated for 16 hours. Infections were performed at an MOI of 10 pfu/cell in M199 lacking FBS. Following a 1.5 h adsorption, the inoculum was replaced with DMEM lacking L-cysteine and L-methionine (Mediatech), and supplemented with 5 μg/mL actinomycin D and 30 μCi/mL Trans ^35^S Label (MP Biomedicals, Inc.), and then incubated for six hours at 37^o^C. Cells were harvested by scraping into PBS, and then centrifuged for 30 seconds at 750 x *g*. Cell pellets were lysed in ECB buffer (120 mM NaCl, 1 mM EDTA, 0.5% Igepal CA-630, 50 mM Tris-Cl pH 8.0) supplemented with protease inhibitor cocktail (Roche Diagnostics). Ten percent of each sample was set aside for input, and the remaining sample was divided evenly and incubated with anti-p65 (Rockland). Antibody-antigen complexes were collected with GammaBind Plus Sepharose (GE Healthcare) according to the manufacturer’s recommendations. The input controls and eluted samples were separated on a 12% acrylamide gel, and proteins were detected by autoradiography.

**A5-16 infections in the presence and absence of MG132 (Figure S4)** To confirm proteasome dependence of IκBα degradation, MA104 cells were infected with A5-16 in the presence or absence of MG132. Lysates were prepared every two hours for ten hours, and immunoblots were probed with anti-IκBα or anti-GAPDH antibodies.

**β-catenin stabilization (Figure S5)**

Rotavirus strains were treated with 10 ug/mL Worthington trypsin and then 293 cells were infected at a MOI of three pfu/cell. The cells were incubated for one hour at 37^o^C in RPMI lacking FBS for one hour, and then FBS was added to 5% (v/v). At ten hpi, cells were harvested in RIPA buffer and used in immunoblot analysis using antibodies for β-catenin (Santa Cruz Biotechnology) and GAPDH. The abundance of β-catenin was determined by immunoblot and densitometry normalized to GAPDH. The level of β-catenin in OSU infected cells was ~2-4 fold higher than in mock infected or A5-16 infected cells.
